# The interplay of DNA repair context with target sequence predictably biasses Cas9-generated mutations

**DOI:** 10.1101/2023.06.28.546891

**Published:** 2023-06-28

**Authors:** Ananth Pallaseni, Elin Madli Peets, Gareth Girling, Luca Crepaldi, Ivan Kuzmin, Uku Raudvere, Hedi Peterson, Özdemirhan Serçin, Balca R. Mardin, Michael Kosicki, Leopold Parts

**Affiliations:** 1 Wellcome Sanger Institute, Wellcome Genome Campus, Hinxton, United Kingdom; 2 Department of Computer Science, University of Tartu, Tartu, Estonia; 3 BioMed X Institute (GmbH), Im Neuenheimer Feld 515, Heidelberg, Germany; 4 Department of Medicine, University of Cambridge, Cambridge, United Kingdom; 5 Lawrence Berkeley National Laboratory, Berkeley, California, USA

## Abstract

The genome engineering capability of the CRISPR/Cas system depends on the DNA repair machinery to generate the final outcome. Several genes can have an impact on mutations created, but their exact function and contribution to the result of the repair are not completely characterised. This lack of knowledge has limited the ability to comprehend and regulate the editing outcomes. Here, we measure how the absence of 21 repair genes changes the mutation outcomes of Cas9-generated cuts at 2,812 synthetic target sequences in mouse embryonic stem cells. Absence of key non-homologous end joining genes Lig4, Xrcc4, and Xlf abolished small insertions and deletions, while disabling key microhomology-mediated repair genes Nbn and Polq reduced frequency of longer deletions. Complex alleles of combined insertion and deletions were preferentially generated in the absence of Xrcc6. We further discover finer structure in the outcome frequency changes for single nucleotide insertions and deletions between large microhomologies that are differentially modulated by the knockouts. We use the knowledge of the reproducible variation across repair milieus to build predictive models of Cas9 editing results that outperform the current standards. This work improves our understanding of DNA repair gene function, and provides avenues for more precise modulation of CRISPR/Cas9-generated mutations.

## Introduction

DNA lesions introduce prompts into the genome that are filled by the repair machinery. As genome editing outcomes are a function of DNA repair, the development of gene editing has revolved around controlling the location of that prompt, and biassing the repair outcomes. The versatile CRISPR/Cas9 technology excels at targeting thanks to its RNA guided nuclease activity, and generates double stranded breaks (DSBs) ^1^. DSBs are the most toxic lesions that a cell can experience, necessitating the evolution of a robust repair response ^2^. This robustness can come at the cost of accuracy, with mutagenic double-stranded break repair leading to the distribution of outcomes seen at Cas9-induced DSBs^3^, while DSB-avoiding Cas9 technologies can limit the range of this distribution ^4^. The generation of loss-of-function mutations has been used to great effect in basic research on genome function and the more precisely controlled technologies for therapeutic purposes to treat disease ^4^.

The stochasticity in repair makes Cas9 a somewhat unpredictable tool. There is substantial variety in the repair outcomes observed within and across targeted sites, both in type and size of mutation generated, while the distribution of outcomes is highly reproducible at each target ^3,5,6^. It is now well understood how the sequence composition of the target site affects the distribution of outcomes generated by Cas9, and computational tools have been developed to accurately predict both the efficacy of cutting as well as outcome distribution at a given target sequence ^7-10^. These tools enable more efficient targeting to create frameshift mutations for knockouts, as well as more precise outcome generation for therapeutic purposes.

The editing outcomes can vary across cell types ^7^, suggesting that there is an avenue for their control that is rooted in repair machinery ^11-13^. Three major repair pathways act on a DSB in mammalian cells. Non-homologous end joining (NHEJ) creates small insertions and deletions, microhomology-mediated end joining (MMEJ) exclusively leaves deletions between short stretches of identical sequence (“microhomology”), and homologous repair (HR) perfectly repairs the break with no mutations ^2^. The pathways are active at different rates and operate in competition with one another ^14,15^, providing redundancy in protection. NHEJ and MMEJ are active throughout the cell cycle, and repair the bulk of DSBs, while HR is only active during S phase ^16,17^. Their contribution to gene editing has so far been tested using a small number of gRNAs ^18-20^, and the roles of many involved genes have not been completely elucidated. Observing editing outcomes in multiple sequence contexts in repair deficient backgrounds would advance understanding of DNA repair genes and mechanisms, and options for control.

Here, we systematically measure the impact of repair gene knockouts on Cas9-generated DSB repair outcomes. We analyse mutations created at 2,812 target sites in 21 mouse embryonic stem cell lines (mESCs), each with a single repair gene knockout. We elucidate how the absence of repair genes modulates Cas9 mutation profiles, associate trends in these profiles with target sequence characteristics, and use this knowledge to build predictive models of repair outcomes for each knockout which outperform existing prediction methods.

## Results

### Measuring Cas9 repair outcomes at scale in knockout cell lines

**Figure 1.**
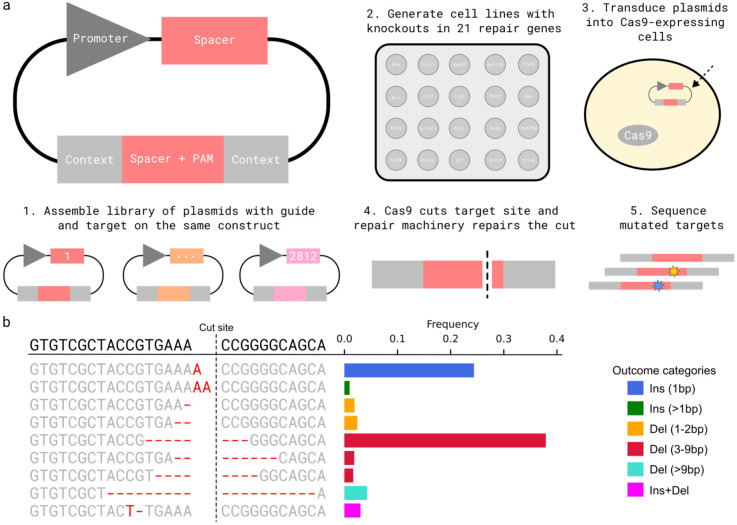
Measuring Cas9 repair outcomes at scale in knockout cell lines **(a)** A method for high throughput measurement of Cas9-induced repair outcomes. (1) Constructs containing both a gRNA and its target sequence (matched colours) in variable context (grey boxes) were cloned into target vectors. (2) A panel of Cas9-expressing mouse embryonic stem cell lines deficient in individual repair genes was generated (Kosicki et al. 2022) (3) Constructs were packaged into lentiviral particles and used to infect the knockout cells. (4) Cas9 cuts the target and mutations are created. (5) DNA from cells was extracted, the target sequence and context are amplified with common primers, and the mutations in the target are determined by short-read sequencing. **(b)** An example mutation distribution for a target. The sequence (left, text) and frequency (x-axis, bars) of each outcome (y-axis). Colours: outcome category. Top sequence: unedited target; vertical dashed line: cut site; red text: altered sequence.

We measured Cas9-generated mutation outcomes at randomly integrated synthetic target sequences within a common sequence context in 21 knockout mouse embryonic stem cell lines (Table 1) and three control cell lines (Figure 1a). After aggregating data from biological replicates, and filtering for coverage, we compiled an outcome distribution for 2,812 target sites in each cell line (Methods, Figure 1b). We recovered 21,341 individual outcomes, for an average of six outcomes per target per cell line. To enable comparisons, we classified outcomes by type and size into seven groups: short deletions (1-2bp), medium deletions (3-9bp), long deletions (>9bp), single insertions (1bp), longer insertions (>1bp) and insertions plus deletions (everything else, Figure 1b).

### Knocking out repair genes modulates outcome profiles

**Figure 2.**
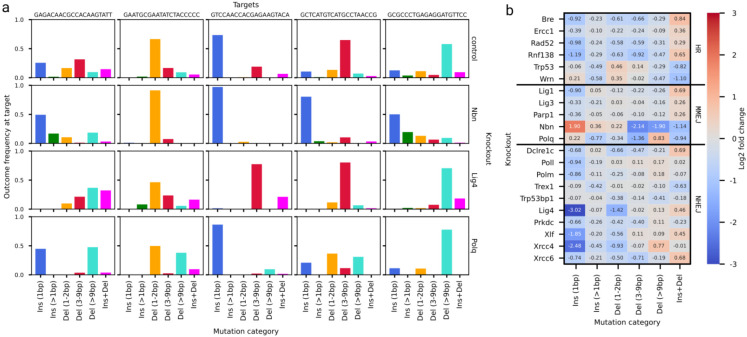
**(a)** Target outcome profiles are consistently modulated by some knockouts. The fraction of mutated reads (y-axis) of each outcome type (x-axis, colours) at various representative targets (columns) in control lines, the Lig4 knockout, the Polq knockout, and the Nbn-knockout (rows). **(b)** Average of log-fold change across all targets (annotation, colour) of each outcome category (x-axis) observed in each knockout (y-axis) organized by repair pathway.

Knocking out repair genes can suppress some outcomes and enrich others (Figure 2a). To quantify knockout effects, we calculated the log-fold change of each outcome category in each target compared to the control, and averaged them for every knockout. These summarised changes were largely consistent with the importance of the gene to its repair pathway and the behaviour of that pathway behaviour.

Lig4 is essential to NHEJ ^35^. Its knockout resulted in suppression of small indels in our screen, especially of otherwise-common single insertions, consistent with the suppression of the NHEJ pathway (Figure 2a). Longer deletions and combinations of insertions and deletions were increased in their place, suggesting that repair was completed by alternative mechanisms. Knockouts of other core NHEJ genes such as Xlf and Xrcc4 had a similar phenotype, suppressing single nucleotide insertions (mean LFC < −1.5, Figure 2b). Other NHEJ genes responsible for narrower aspects of end-processing such as Dclre1c, Poll, and Polm had a smaller effect (mean LFC between −0.5 and −1). Interestingly to us, the Xrcc6 knockout also only produced a mild suppressive effect despite the importance of the Ku dimer to the NHEJ process (Figure 2b).

MMEJ knockouts were less consistent in their effects. Polq is core to MMEJ, and its knockout suppresses the formation of medium length deletions (mean LFC of −1.4, Figure 2b), but not larger ones (mean LFC of 0.8). Medium deletions are emblematic of MMEJ ^33,36^, and the profiles missing these outcomes are consistent with complementation by repair pathways that perform longer resection, such as homology-directed repair or single strand annealing. Nbn is also involved in MMEJ and produced the strongest effect of all tested genes in the panel. Its knockout suppressed deletions of all sizes (mean LFC of −2.1 for medium deletions, −1.9 for long deletions, Figure 2b) and resulted in profiles enriched in small mutations, especially 1bp insertions (mean LFC of 1.9). Nbn is part of the MRN complex, involved in medium and long resection as part of MMEJ, HR and single strand annealing ^27,37^, and profiles in its knockout are consistent with the inability of the cell to perform long resection and thus having to rely on NHEJ. The other MMEJ-associated genes in our panel (Lig1, Lig3 and Parp1) had less striking effects and were not aligned with the behaviour of either Polq or Nbn (Figure 2b).

The remaining knockouts were not consistent in their effect, with multiple interesting phenotypes shared across repair pathways, or specific to subsets of a single one. Knockouts of the HR genes could be split into two groups. Bre, Ercc1, Rad52 and Rnf138 knockouts were enriched in complex outcomes (mean LFC > 0.2, Figure 2b), while these outcomes were depleted in Trp53 and Wrn (mean LFC < −0.8). A similar increase in complex outcomes was present in knockouts of Lig1, Dclre1c, and Xrcc6 (mean LFC > 0.6). We speculate that these effects stem from the role of these genes in DNA resection coupled with ligation-derived insertions.

### Variable response to knockouts distinguishes mutation classes

**Figure 3.**
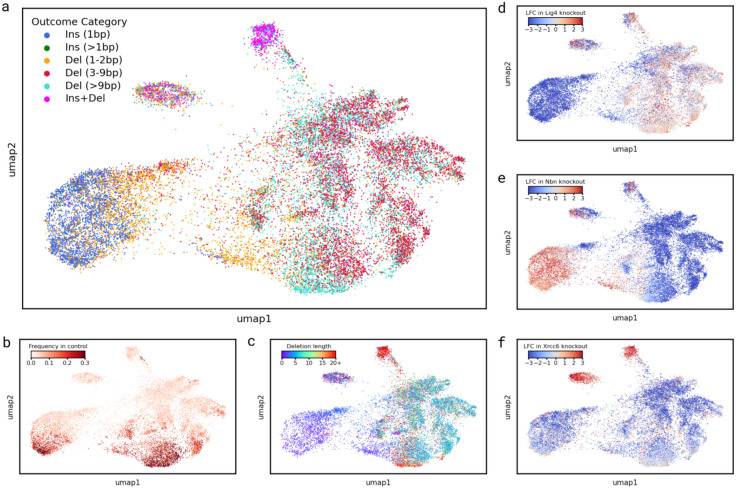
UMAP embedding of outcome modulation profiles, coloured by: **(a)** outcome category, **(b)** frequency of outcomes in the combined control line, **(c)** number of deleted nucleotides in the outcome, **(d)** log2-fold change of outcome frequency in the Lig4 knockout, **(e)** log2-fold change of outcome frequency in the Nbn knockout, **(f)** log2-fold change of outcome frequency in the Xrcc6 knockout.

To understand how primary DNA sequence of gRNA targets determines DNA damage repair outcomes, we embedded the log2-fold changes of 21,341 individual repair outcomes across the 21 knockout lines into two dimensions using UMAP (Figure 3a). Distance between two outcomes in this representation reflects the similarity of log-fold-change in their frequencies across knockout lines, and indicates shared response to repair modulation.

Outcomes of the same category or similar frequency and size tended to co-localize within the embedding (Figure 3a-c). Over 90% of all 1bp insertions formed a single cluster, interspersed with about 50% of small deletions (1-2bp), all of which were depleted in the Lig4 knockout line (Figure 3d). The remaining small deletions formed part of the largest cluster, which was otherwise composed primarily of medium and large deletions (3bp+). That cluster also contained a distinct section composed of the majority of frequent and large deletions (>20nt; Figure 3b,c). Most medium and large deletions were depleted upon knocking out Nbn (Figure 3e). Remaining outcomes formed two smaller clusters, both strongly associated with Xrcc6 (Figure 3f). One of these clusters was strongly enriched for rarer insertion-deletions, while the other contained a mix of different outcome categories. Most clusters contain finer structure, which we explore in the following sections to tease out the DNA sequence dependencies of DSB repair.

### Duplications of PAM-distal base are dependent on Polm-Prkdc action

**Figure 4:**
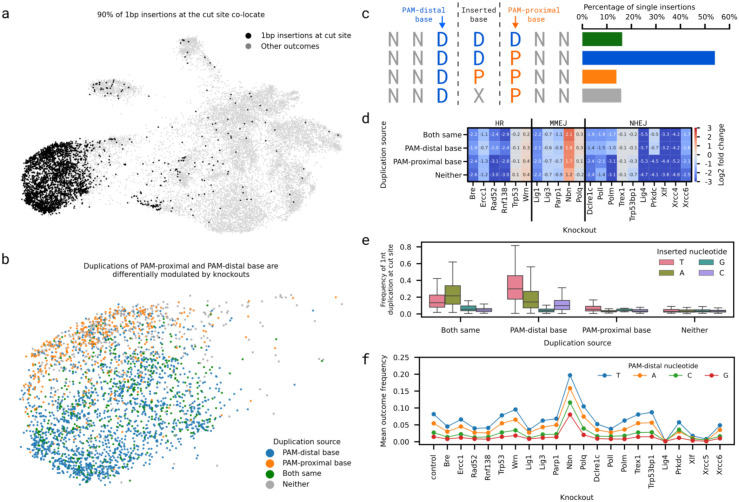
**a.** 90% of single insertions at the cut site co-localize. UMAP embedding (x- and y-axis) of outcome modulation profiles (markers). Black: 1bp insertions; grey: all other outcomes. **b.** Insertions at the cut site segregate based on the source of the duplicated nucleotide. Subset of UMAP embedding from (a) containing 90% of single insertions at the cut site. Blue: PAM-distal base inserted; orange: PAM-proximal base inserted; green: PAM-distal and PAM-proximal bases identical; grey: inserted base neither PAM-distal nor PAM-proximal. **c.** Most single insertions at the cut site were duplications of the PAM-distal base. Percentage of single insertions at the cut site (x-axis) for four sources of inserted nucleotide (y-axis), with PAM-distal (blue) and PAM-proximal (orange) nucleotides highlighted around the cut site (dashed line). **d.** Duplications of the PAM-proximal nucleotide were depleted in the absence of Prkdc and Polm, while PAM-distal duplications were not affected. Log-fold change (annotation, colour) of cut site duplications in each knockout (x-axis) based on the source of the duplicated nucleotide (y-axis). **e.** Adenosines and thymines were enriched in 1bp insertions. Distribution of outcome frequency (y-axis) of duplications at the cut site for each source of the duplicated base (x-axis) split by the identity of the duplicated nucleotide (colours). **f.** Preference for adenosines and thymines is consistent across knockouts. Mean frequency (y-axis) of duplications at the cut site split by identity of the duplicated base (colours) in each knockout line (x-axis).

Insertions of 1bp could arise by non-templated addition to a blunt DSB, from fill in of a 5' overhang DSB, or due to templated addition in *trans*, e.g. from the opposite side of the DSB. While the exact provenance of any 1bp insertion cannot be established with certainty, the identity of the nucleotides flanking the cut site provides important clues. Over 90% of 1bp insertions at the cut site observed in the screen clustered together (Figure 4a), and over 80% matched one of the nucleotides flanking the cut site (43% expected, Figure 4b-c). Duplications of the PAM-distal nucleotide were the most prevalent among these, consistent with the finding that Cas9 can generate DSBs with 1nt 5' overhang, which can be filled in to create 1bp templated insertions ^38,39^. Duplications were enriched for adenosines and thymines (Figure 4e), which is consistent throughout the knockouts (Figure 4f; ^40^).

Duplications of the PAM-proximal nucleotide were separated in the embedding from duplications of the PAM-distal nucleotide, indicating different mechanisms were responsible for their creation. The former were severely depleted in Prkdc or Polm deficient cells (LFC < - 4 and LFC < −3, respectively), while the latter were mildly affected (LFC > −1, Figure 4d). This difference could be due to Prkdc-Dclre1c mediated 5' resection of the distal end of a blunt DSB, revealing 3' overhang substrate for Polm (see Discussion; ^41^). Consistent with this, PAM-proximal duplications were also more depleted in Dclre1c deficient cells than PAM-distal ones, although the difference was smaller (LFC −2.4 and −1.4) than in the case of Prkdc and Polm.

### Deletions between large microhomologies are dependent on Nbn but not Polq

**Figure 5.**
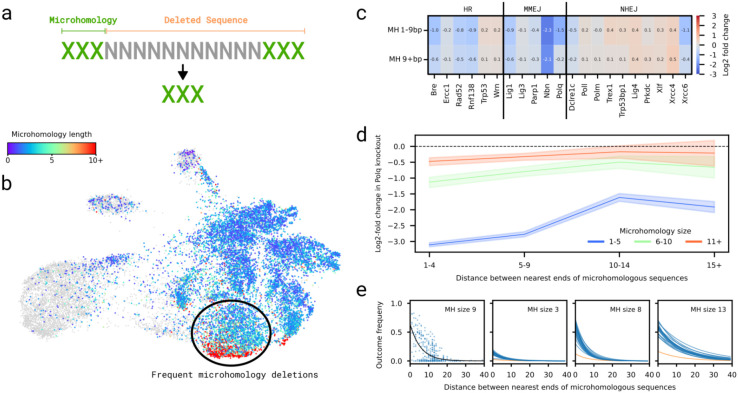
**(a)** Illustration of a microhomology deletion. Microhomology deletions are a deletion of sequence between two homologous sequence regions, including the loss of one of the regions. **(b)** Deletions utilising microhomologies larger than 10bp cluster together. UMAP embedding of outcomes coloured by size of microhomology involved in deletions. **(c)** Frequent deletions involving longer microhomologies are separated from other deletions in the cluster by less depletion in the absence of Polq. Mean log-fold change relative to the control line (annotations, colours) of deletions involving microhomology for microhomologies less than 9bp long and larger than or equal to 9bp long (y-axis) in each knockout (x-axis). **(d)** Shorter and closer microhomologies are more dependent on Polq. Log2-fold change of deletion outcomes in the Polq knockout (y-axis) based on the distance between nearest ends of microhomology sequences used in the deletion (x-axis) for each size of microhomology sequence (colour). Bands indicate 95% confidence intervals. **(e)** We model the relationship between outcome frequency, size of microhomology sequence, and distance between microhomology sequences and find it consistent between knockouts except Nbn. Frequency of microhomology deletion (y-axis) vs the distance between microhomology sequence (x-axis) for a single length of microhomology (panels) for each knockout (blue lines), with Nbn highlighted (orange line). First panel shows the data points used to train models for a single knockout and microhomology size.

The majority of Lig4-invariant outcomes were medium and long deletions, which tend to co-localize in the embedding (Figure 3). Over 90% of these occurred between two regions of microhomology (Figure 5a), and were depleted in the absence of Nbn. Deletions involving microhomologies of 10bp or greater clustered together (Figure 5b) and were invariant to the absence of Polq (mean LFC of 0.2 vs −1.5 for shorter ones, Figure 5c). These longer microhomology deletions were frequent (mean outcome frequency in control of 0.44 for microhomologies of 10 or larger, Figure 5b, 3b) and strongly affected by only one knockout, Nbn (Figure 5c). For all microhomology mediated deletions, the size of microhomology sequences and the distance between them determined the dependence of that outcome on Polq. Smaller and closer microhomologies were more dependent on Polq, while larger and further microhomologies were less dependent (Figure 5d). These observations suggest that Polq alone enables the utilisation of shorter tracts of microhomology that are within 10-14bp of each other, but deletions involving microhomologies of at least 10bp or between microhomologies that are further apart are created by a Polq-invariant mechanism.

We next asked whether the repair gene knockouts modulate the quantitative characteristics of microhomology-mediated deletion formation. To do so, we modelled the frequency of deletions as a function of microhomology length and distance between the ends of the microhomologous sequences (Methods). Deletions utilising longer and closer microhomologies are more likely to occur than those using shorter or more distant ones ^7^. These trends are almost invariant across knockouts (Figure 5e, Supplementary Figure 1), with the exception of Nbn, which exhibited an attenuated trend. We speculate that the increased affinity of longer microhomologies lends itself more readily to direct annealing of the broken ends after resection and needs less assistance from Polq.

### Xrcc6, Rnf138 and Rad52 modulate complex outcomes and deletions

**Figure 6:**
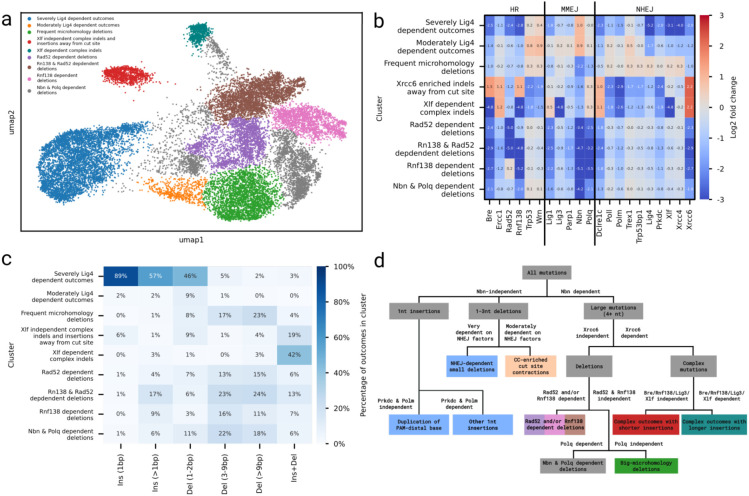
**a.** UMAP embedding of outcome modulation profiles coloured by proposed cluster identities. **b.** Outcomes in clusters have distinct modulation profiles. Average log-fold change (annotation, colours) of outcomes in each cluster (y-axis) for each knockout line (y-axis). **c.** Distribution of outcomes among clusters. Percentage (colour) of each outcome category (x-axis) present in each cluster (y-axis). Each column sums up to 100%. For distribution of clusters among outcome categories, see Supplementary Figure 5. **d.** Flowchart of main knockout sensitivity for the outcome categories identified in this study.

Unbiased clustering of outcomes in UMAP space revealed two very distinct groups that contained the majority of complex insertion-deletion outcomes and were rare in control cells (Figure 6a, 3b). Both were the only groups strongly enriched in the absence of Xrcc6 (mean LFC > 2) and, to a lesser degree, in the absence of Ercc1 or Dclre1c (mean LFC >= 1), which implies failure of end protection or short distance resection. At the same time, these clusters were strongly depleted in Prkdc, Polm and Poll knockouts, implying they both rely on addition of nucleotides. The two clusters were very distinct from each other in terms of both their reliance of DNA damage machinery and types of outcomes produced. One of the clusters was strongly depleted by Bre, Rnf138, Lig3 and Xlf, and consisted primarily of long deletions (>20bp; Figure 2c) with long insertions (mean length 6.2bp vs 3.7bp for other sub-group), many of which were distal to the cut site (Supplementary Figures 2 and 3). In contrast, the other cluster was either invariant to or mildly enriched by these deficiencies, and almost entirely composed of small cutsite-distal outcomes (1-2bp; Supplementary Figures 2 and 3).

Our clustering also revealed a substructure among medium and large deletion outcomes (Figure 6c). These deletion clusters were differentiated from each other by differential depletion in response to the Polq knockout (Supplementary Figure 4) and by severe depletion in either the Rad52 knockout, the Rnf138 knockout, both, or neither (Figure 6a,b). These observations suggest a more nuanced involvement of the relevant genes in their pathways and warrant further study to determine their mechanisms.

Finally, we synthesised the observations above into a set of top-level clusters that describe the major modulations of the outcomes in the embedding (Figure 6a). These clusters differ from each other based on knockout effect (Figure 6b), and encompass the full variety of outcomes that we observed in the screen (Figure 6c, Supplementary Figure 5). An interactive version of this visualisation, as well as more complex clustering is available at https://partslab.sanger.ac.uk/FORECasT-BE/umap/. The organisation of the outcomes by similarity to knockout effects can be succinctly captured in a tree (Figure 6d) that reflects the major outcome categories and their dependence on the repair pathways.

### Outcome profiles are predictable in knockout contexts

**Figure 7.**
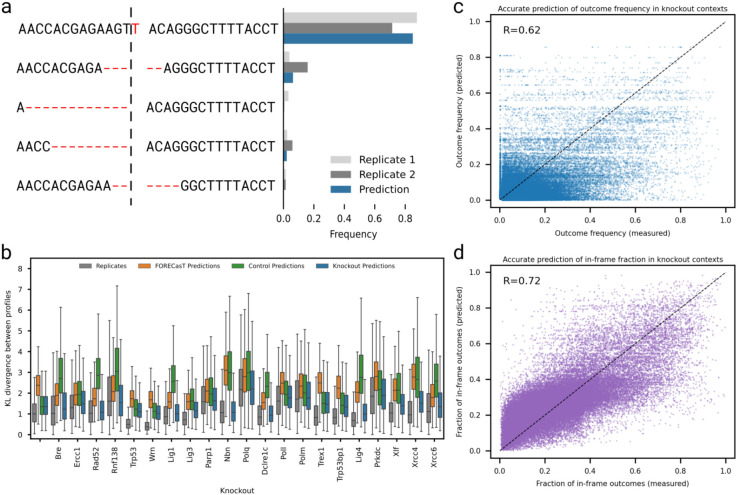
**a.** Example of outcome profile prediction for a single target in a knockout line. Fraction of mutated reads (x-axis) for each outcome (y-axis) measured in replicates (light grey and dark grey bars) and predicted by our model (blue bars). Green boxes = microhomology; grey boxes = deletion; dashed line = cut site. **b.** Distribution of KL divergences between outcome profiles in the same target (y-axis) in each knockout line (x-axis) when comparing replicates (grey), measured frequencies to FORECasT predictions (orange), measured frequencies to predictions from the control model (green), and measured frequencies to knockout model predictions (blue). **c.** Measured outcome frequencies (x-axis) and predicted outcome frequencies (y-axis) for outcomes in held-out targets in all knockouts. Label: Pearson's R between measured and predicted frequencies. **d.** Accurate prediction of frameshift outcomes. Measured fraction of in-frame outcomes (x-axis) and predicted fraction of in-frame outcomes (y-axis) for held-out targets across all knockouts. Label: Pearson's R between measured and predicted in-frame frequencies.

Having quantified the sequence determinants of Cas9 outcomes in repair-deficient contexts, we set out to build predictive models of their behaviour. We used the FORECasT model ^7^, a multiclass regression that predicts the frequency of individual outcomes generated at a target from sequence features (Figure 7a). We split the data into training and test sets, and trained one FORECasT model per knockout, using Kullback-Leibler (KL) divergence between the predicted outcome profile and the measured ones as quality metric. The trained model is available at https://github.com/ananth-pallaseni/FORECasT-repair and as a web tool at https://elixir.ut.ee/forecast-repair/. The distribution of KL divergences from predictions was close to the one observed between replicates (average divergence 0.1 vs 0.2 between replicates; Figure 7b). Knockout specific models also outperformed the control model and the original FORECasT model on their lines, especially on lines with strong modulation phenotypes like the Nbn knockout (average divergence 0.1 vs 1.3 for control line and 1.1 for FORECasT; Figure 7b).

## Discussion

We presented the largest assessment of Cas9-induced outcomes in repair deficient contexts to date. The absence of repair genes can strongly modulate the frequency of outcomes generated by DSB repair, often consistent with their primary repair pathway. NHEJ knockouts suppressed the generation of small indels, while the absence of Polq suppressed medium length deletions, and the Nbn knockout was depleted for both medium and long deletions. Complex outcomes involving both insertions and deletions were enriched in the absence of Xrcc6.

We have observed a strong depletion of PAM-proximal nucleotide templated insertions in Polm and Prkdc deficient cell lines. We speculate that they may arise when DNA-PKcs (product of Prkdc) stimulates Artemis (product of Dclrce1) to resect 1nt of the 5' end of a blunt DSB, revealing a 3' overhang. This process would occur more efficiently on the distal end of the DSB, which is not 'protected' by Cas9 binding. The 3' overhang would then be used as a substrate by Polm twice in a row ^41^ - once to 'restore' the resected nucleotide on the other side of the break (creating a perfect 1nt 3' overhang match that if ligated would restore wild-type sequence) and a second time to add the 'insertion' nucleotide.

The assay we used has some limitations. Redundancy of function, especially in the robust NHEJ pathway, is a confounder of all single knockout effects. While consistent modulation of outcomes indicates a causal link, limited signal is not necessarily evidence of a lack of participation in the DSB repair process. For example, both Poll and Polm in the NHEJ pathway can perform the required nucleotide synthesis for end processing on certain substrates ^30^. In addition, gene-specific sequence features that dictate outcomes could be missing in the library of ~2,800 targets. A logical next step to further improve understanding is therefore to perform screens in contexts where pairs of genes are perturbed using larger target libraries. Finally, the assay is limited to measuring outcomes primarily generated by NHEJ and MMEJ, and the effect of HR-associated genes could only be viewed through their modulation of these outcome types. It is known that the rate of HR in mammalian cells is affected by the absence of Lig4 ^42^ and it is likely that other genes in the panel also have an effect. Future studies into DSB repair using large scale screens could consider the integration of HR-reporters to improve our understanding of this process.

The activity of repair pathways is the key confounder of all gene editing experiments. The data and models presented in this study shed light on the nature of these interactions for 21 proteins in the cast of DSB repair, but the behaviours and dependencies of the rest remain largely unknown. Similar screens using larger target panels in combinatorial knock-out contexts are needed to cover all relevant repair pathways, and to understand this complex process to predict its results *a priori*. Accurate predictions combined with options to modulate repair will enable fine control over the outcomes of Cas9-based genome editing.

## Methods

### Library cloning

To generate the library, a 197-mer oligo pool encoding 5,760 oligonucleotides was ordered from Twist Bioscience. The library was amplified by PCR using KAPA HiFi HotStart ReadyMix (Roche), 2 ng of template, 0.15μM subpool forward primers P1 or P2 and 0.15 μM universal reverse primer P3. To reduce the number of polymerase induced mistakes, 10 cycles were used for the PCR. A nested PCR to add GIbson homology ends was done with KAPA polymerase, 0.15 μM primers P4 and P3 over 10 cycles using 2 ng of template DNA. After each PCR, amplicons were purified using Monarch^®^ PCR & DNA Cleanup Kit (NEB).

A lentiviral gRNA expression vector lacking the scaffold, pKLV2 - U6(BbsI) - PGKpuro - 2A - mCherry - W, was generated by removing the improved gRNA scaffold from pKLV2 - U6 - gRNA5(BbsI) - PGKpuro - mCherry - W (Addgene 67977) (^7^ with minor changes). The amplicons were cloned into the vector using Gibson Assembly mix reactions (NEBuilder^®^ HiFi DNA Assembly Cloning Kit) according to manufacturer’s specifications in two or three separate reactions. Gibson reactions were pooled, column-purified and transformed in 4 or 5 electroporations (NEB 10-beta Electrocompetent E. coli C3020K) for a coverage of more than 425x. Bacterial cells were cultured overnight in liquid and plasmid DNA encoding an intermediate library was extracted using QIAGEN Plasmid Maxi Kit (QIAGEN). The vectors were digested with BbsI (NEB).

A 221-mer G-block (IDT) encoding the improved scaffold was amplified using 5 ng of template, KAPA polymerase, 0.1 μM primers P5 and P6 over 25 cycles ^7^. The product was column-purified with Monarch^®^ PCR & DNA Cleanup Kit (NEB) and digested with BbsI. The intermediate library and G-block were column-purified and ligated (T4 DNA ligase, NEB) in three separate reactions for each subpool. The reactions were combined and digested again with BbsI 37°C for 30 min to remove any undigested carryover products. The products were column-purified and transformed in either 4 or 5 electroporations. Bacterial cells were cultured overnight in liquid and the final libraries were extracted using QIAGEN Plasmid Maxi Kit (QIAGEN). The libraries were quantified and subpools combined in 1:4.76 molar ratio to get the final library containing 5,760 gRNAs.

### Cell culture

CAST/BL6 (CB9) mouse embryonic stem cells (mESC) that expressed Cas9 and had a knockout of a gene in the DNA repair pathway ^20^, were cultured in M15 media (high-glucose DMEM (Lonza), with 15% FCS (ThermoFisher), 0.1 mM beta-mercaptoethanol,100 U/ml penicillin and 100 mg/ml streptomycin (Gibco)) on SNL-HBP feeder cells. Cells were treated with 10 μg/ml blasticidin for at least 3 days before starting a screen to ensure stable Cas9 expression. The screens were performed without feeder cells in M15 medium supplemented with 1000 U/ml leukemia inhibitory factor (Merck). Cells were plated on flasks coated with 0.1% gelatin solution (Merck). Medium was changed daily throughout expansion and all experiments. All cell lines were cultured at 37°C, 5% CO2.

### Lentivirus production and determination of lentiviral titer

Supernatants containing lentiviral particles were produced by transient transfection of 293FT cells using Lipofectamine LTX (Invitrogen). 5.4 μg of a lentiviral plasmid library, 5.4 μg of psPax2 (Addgene 12260), 1.2 μg of pMD2.G (Addgene 12259) and 12 μl of PLUS reagent were added to 3 ml of OPTI-MEM and incubated for 5 min at room temperature. 36 μl of the LTX reagent was then added to the mixture and further incubated for 30 min at room temperature. The transfection complex was added to 80%-confluent 293FT cells in a 10-cm dish containing 10 ml of culture medium. After 48h viral supernatant was harvested and fresh medium was added. After 24h the lentiviral supernatant was collected, pooled with the first supernatant, filtered through a 0.45μm filter and stored at −80 °C.

For lentiviral titration, mES cells were plated into 96-well plates, 5x10^4^ cells per well. 8 μg/ml Polybrene (hexadimethrine bromide, Sigma) was added to each well and the cells were transduced with varying volumes of virus (0 to 20 μl). The cells were then centrifuged at 1000g for 30 minutes at room temperature and resuspended in the same media. After three days of cell culture, cells were harvested for FACS analysis and the level of mCherry expression was measured. Data was analysed with Flowjo. Virus titer was estimated and scaled up accordingly for subsequent screens.

### Screening of repair outcomes

mES cell lines were infected aiming for a multiplicity of infection (MOI) of 0.6 to 0.8 and at a coverage 800x. The effective MOI ranged between 0.1-0.6 with a coverage of 100-650x depending on the cell line. For each line, at least two infections were performed and treated as separate biological replicates. Cells were seeded onto 0.1% gelatin coated flasks with a density of 3x10^4^ cells/cm^2^. 24 h after transduction 3 μg/ml of puromycin was added and maintained throughout the screen. Cells were cultured for 14 d after infection. Samples were taken on day 3, 7, 10 and 14 post-infection. Enough cells were passaged and collected to maintain coverage higher than at the time of infection.

### DNA extraction and sequencing library preparation

Upon collection, cells were centrifuged and pellets were stored at −20°C. For genomic DNA extraction, cell pellets were resuspended into 100 mM Tris-HCl, pH 8.0 (Thermo Scientific), 5 mM EDTA (Invitrogen), 200 mM NaCl (Invitrogen), 0.2% SDS (Promega) and 1 mg/ml Proteinase K (Merck) and incubated at 55°C overnight. The solution was treated with 10 μg/ml RNase A for 4 hours. DNA was extracted by adding one volume of isopropanol followed by spooling, double wash with 70% ethanol and elution in TE buffer overnight. DNA was quantified in triplicate using Quant-iT Broad Range kit (Invitrogen).

For sequencing, the region containing the target surrounded by the context was amplified by PCR using primers P7-P8 with Q5 Hot Start High-Fidelity 2× Master Mix (NEB) with the following conditions: 98°C for 30s, 20 cycles of 98°C for 10s, 50°C for 15s and 72°C for 20s, and the final extension 72°C for 5 min. For each sample, the amount of input gDNA template was adjusted to the screen coverage based on measured MOI and ranged from 35 to 103 μg, aliquoted into 50μl reactions each containing no more than 5μg gDNA ^7^. The PCR products were pooled in each group and purified using QIAquick PCR Purification Kit (Qiagen). Sequencing adaptors were added by PCR enrichment of 1 ng of the purified amplicons using forward primer P9 and indexing reverse primer P10 with KAPA HiFi HotStart ReadyMix with the following conditions: 98°C for 30s, 12–16 cycles of 98°C for 10s, 66°C for 15s and 72°C for 20s, and the final extension 72°C for 5 min. The PCR products were purified with Agencourt AMPure XP beads. Samples were quantified with Quant-iT^™^ 1X dsDNA HS Assay (Invitrogen) and sequenced on Illumina HiSeq2500 or HiSeq4000 by 100-bp paired-end sequencing using Illumina standard primers.

### Data processing

Sequencing reads were converted into outcome profiles for each guide using the custom pipeline described in Allen et al ^7^, which assigns reads to guides and uses a dynamic programming approach to identify mutations. Guide profiles with less than 100 reads in any knockout, replicate or timepoint were removed from the analysis to ensure adequate coverage. Outcomes only observed in a single read across all samples were removed. As a result of the filtering, 2,812 guides were retained. Two biological replicates for each knockout at each timepoint were combined by pooling together all the reads assigned to the same guide and treating them as one outcome profile. The three timepoints were combined for each sample in the same fashion as the replicates, as the correlation between them was high.

### Clustering

All clustering analyses were performed using outcomes from a set of 2,812 targets common to all knockouts. Outcomes not present in the corresponding target in the control cell line or not present in at least 5 knockout lines were removed. UMAP projection of outcome modulation profiles was performed using the umap-learn python package ^43^ with a min_distance of 0 and a num_neighbors of 50.

### Modelling microhomology dynamics

To model the relationship between outcome frequency (*y*), microhomology size (*s*) and distance between microhomologies (*d*), we fit exponential models of the form *y*_s_ = *Ae*^*Bd*^) for every size of microhomology from 2 to 15 using the *curve_fit* function in the scipy python package ^44^.

### Modelling outcome frequency

A set of possible outcomes and features was generated for each target using the methodology laid out in Allen et al ^7^. Generated outcomes included every insertion of up to two nucleotides within 3 nucleotides of the cut site and all deletions of up to 30 nucleotides which span the cut site. 3,633 binary features were computed for each outcome, describing their length, location, inserted sequence, involvement of microhomology, and nucleotide context, as well as pairwise combinations of these features. These features were paired with the measured frequencies of the generated outcome in our screens. 0.5 reads were added to each outcome for numerical stability. A dataset of generated outcomes, their scaled frequencies in our experiment, and their corresponding features was produced for each target present in each knockout line. These datasets were each randomly split into a training and test set, keeping 10% of the data in each test set. A logistic regression to predict each outcome in a profile was trained by minimising the KL divergence between the predictions of outcomes in a profile and the measured frequencies for all targets in the training set as in ^7^. Model performance was evaluated by calculating the average KL divergence between measured and predicted profiles in each test set.

## Data availability

### Sequencing data:

European Nucleotide Archive (https://www.ebi.ac.uk/ena/browser/home) accession PRJEB12405

### Processed data:

https://figshare.com/s/6d607804b25f006a72dc

## Supplementary Material

Supplement 1

## Figures and Tables

**Table 1.** The panel of DNA repair gene knockouts, organised by pathway

Knockout	Pathway	Function during DSB repair
Bre	HR	Part of the BRCA1-A complex, responsible for sequestering BRCA1 away from DSB repair sites.^21^
Ercc1	HR	Removes non-homologous 3′ single-stranded tails in HR and NHEJ. Also active in nucleotide excision repair and interstrand crosslink repair. ^22^
Rad52	HR	Promotes HR by displacing RPA from resected ends, allowing Rad51 to bind and start homology search.^23^
Rnf138	HR	Promotes HR activity by displacing Ku.^23^
Trp53	HR	Multifunctional tumour suppressor gene primarily inducing cell cycle arrest and apoptosis in response to DNA damage.^24^
Wrn	HR	Helicase and exonuclease, promotes NHEJ by suppressing resection.^25^
Lig1	MMEJ	Ligates broken ends in MMEJ.^26^
Lig3	MMEJ	Ligates broken ends in MMEJ. Also essential for mitochondrial DNA repair.^26^
Parp1	MMEJ	Binds to resected DNA ends and biases pathway choice towards MMEJ.^15^
Nbn	MMEJ	Part of the MRN complex which performs resection of DSBs, initiating MMEJ or HR.^27^
Polq	MMEJ	Encodes Polymerase Theta, which displaces RPA from ssDNA, anneals sequences with microhomology and adds untemplated and templated nucleotides to the ends of DSBs.^28^
Dclre1c	NHEJ	Encodes Artemis, an endo- and exonuclease active during NHEJ.^29^
Poll	NHEJ	Polymerase. Prefers substrates with complementary sequence between ends. Also active in MMEJ.^30^
Polm	NHEJ	Polymerase. Prefers downstream base as template.^30^
Trex1	NHEJ	A 3’ to 5’ exonuclease. Essential for ssDNA-mediated gene editing.^31^
Trp53bp1	NHEJ	Protects broken ends from resection by MRN complex and helps recruit other repair factors.^32^
Lig4	NHEJ	Primary ligase for NHEJ. Forms a synaptic complex with Xlf and Xrcc4 to hold broken ends together.^33^
Prkdc	NHEJ	Encodes DNA-PKcs, a kinase that binds to Ku complex and enables end processing by Artemis (product of Dclre1c). Regulates cell cycle arrest in response to DSBs.^34^
Xlf	NHEJ	Forms a synaptic complex with Lig4 and Xrcc4 to hold broken ends together.^33^
Xrcc4	NHEJ	Forms a synaptic complex with Lig4 and Xlf to hold broken ends together. Stimulates activity of Lig4 for ligation of broken ends.^33^
Xrcc6	NHEJ	Encodes Ku70, one half of the Ku heterodimer. Senses and binds to broken ends, forming a scaffold for other NHEJ proteins.^33^
