# Supplementary material for: The interplay of DNA repair context with target sequence predictably biasses Cas9-generated mutations
Source: bioRxiv. 2023 Jun 28:2023.06.28.546891. Preprint. [Version 1] doi: 10.1101/2023.06.28.546891 (PMC10326969; doi:10.1101/2023.06.28.546891)
Supplement: Supplementary file 1 [file media-1.pdf]

Supplementary Figure 1

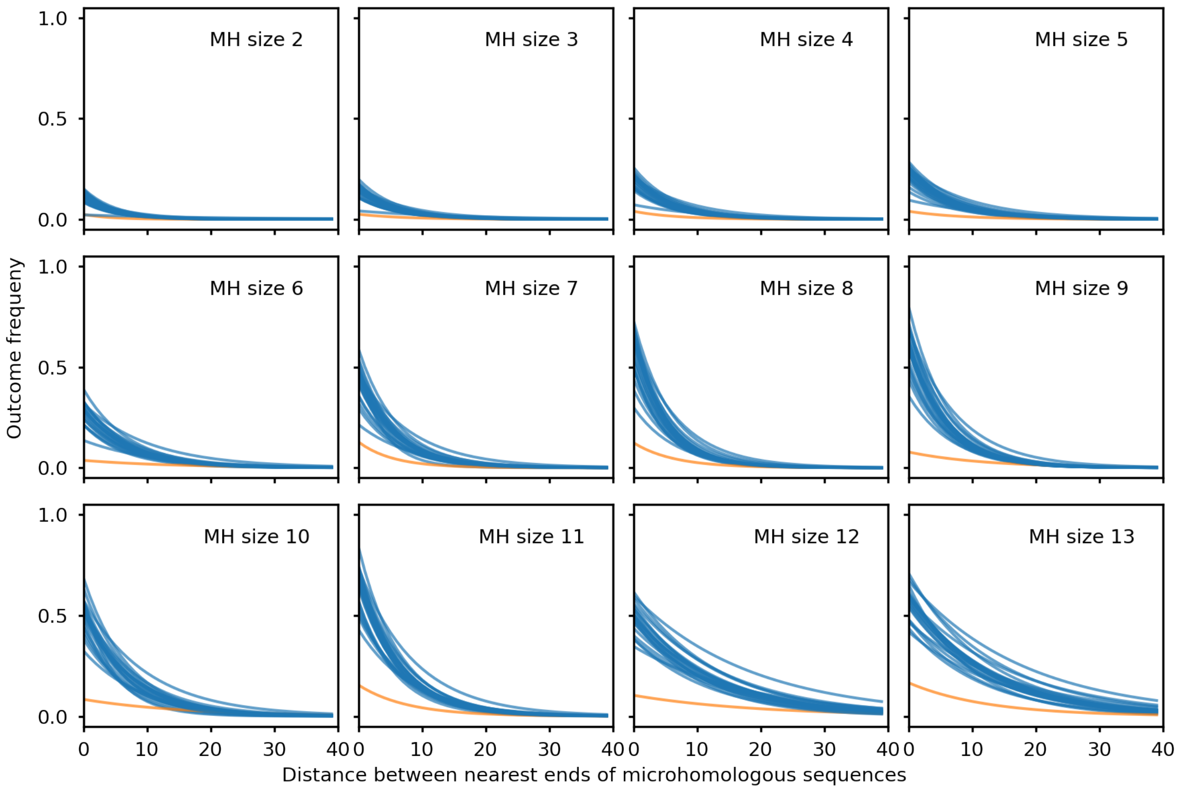

Supplementary Figure 1. We model the relationship between outcome frequency, size of microhomology sequence, and distance between microhomology sequences and find it consistent between knockouts except Nbn. Frequency of microhomology deletion (y-axis) vs the distance between microhomology sequence (x-axis) for a single length of microhomology (panels) for each knockout (blue lines), with Nbn highlighted (orange line).

Supplementary Figure 2

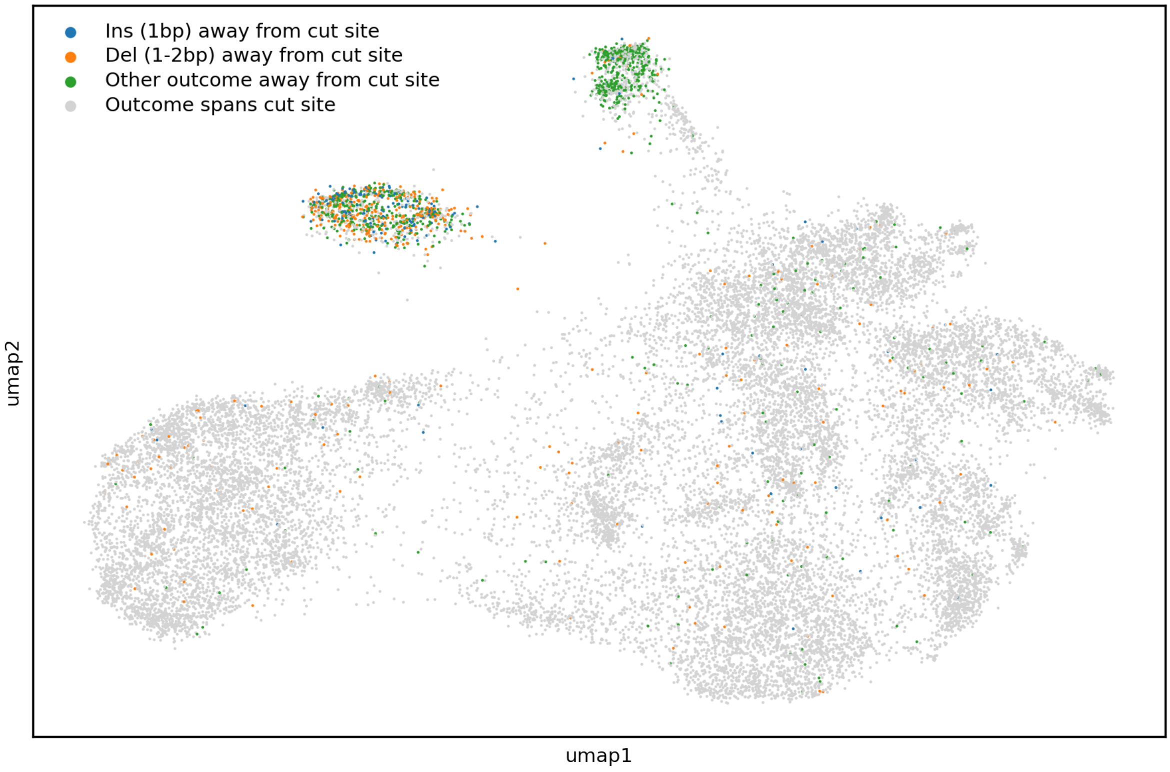

Supplementary Figure 2. Majority of outcomes away from the cut site co-locate into two clusters. UMAP projection of outcomes coloured by category and location (colour) of each outcome (dot).

Supplementary Figure 3

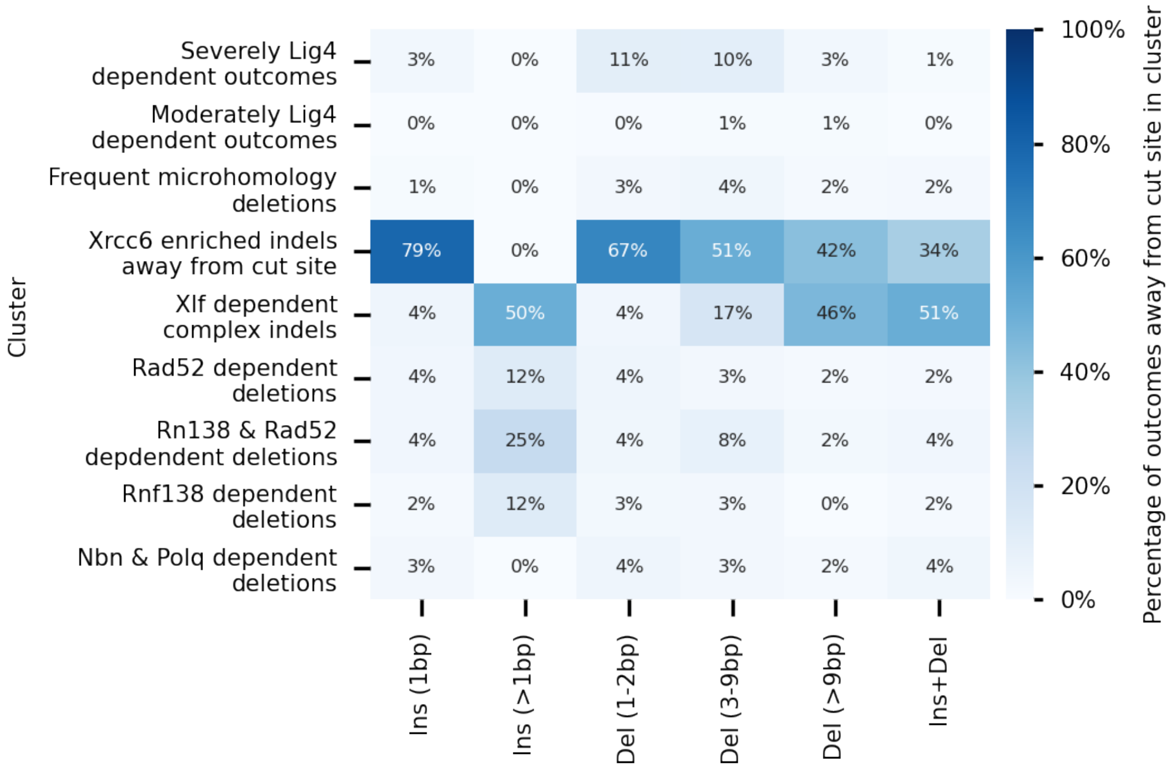

Supplementary Figure 3. Distribution of cut site-distal outcomes, broken down by outcome categories (x-axis), among UMAP clusters (y-axis). Each column sums to 100%.

Supplementary Figure 4

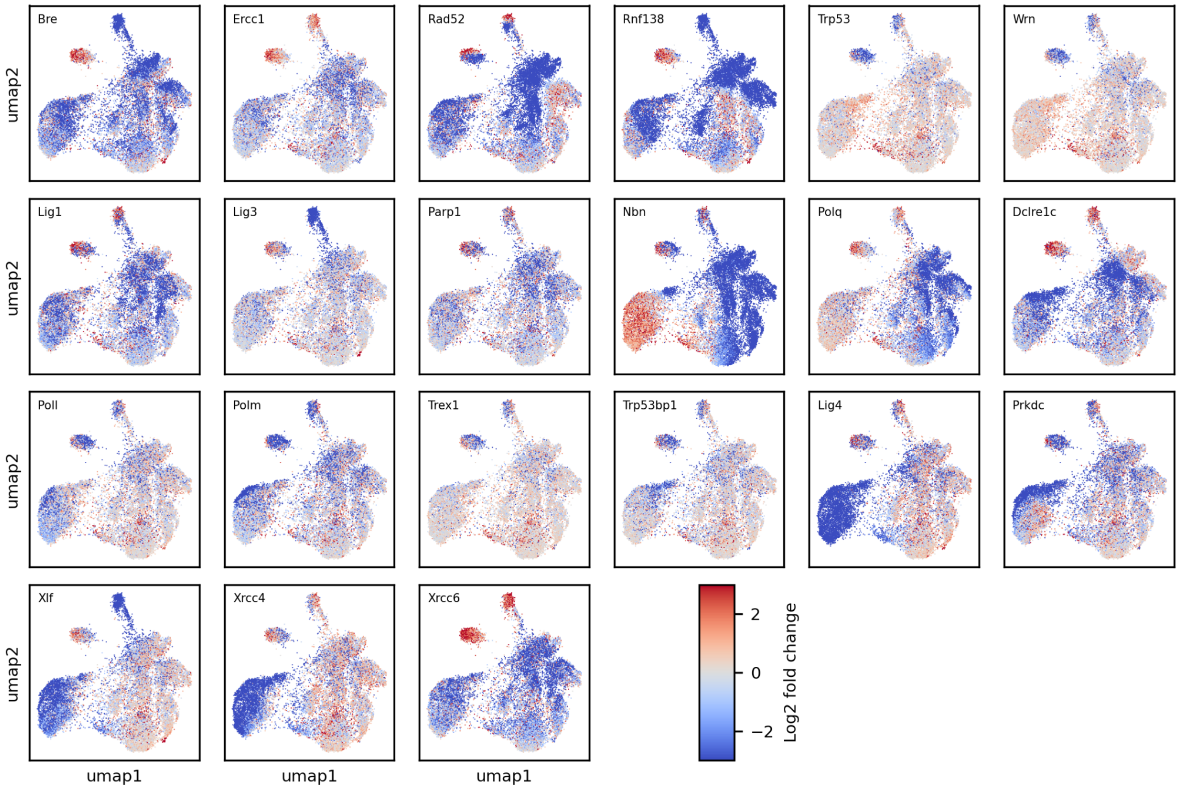

Supplementary Figure 4. UMAP projection of outcomes coloured by log-fold change in each knockout. UMAP projection of outcomes coloured by log-fold change (colour) of each outcome (dot) in each knockout (panels).

Supplementary Figure 5

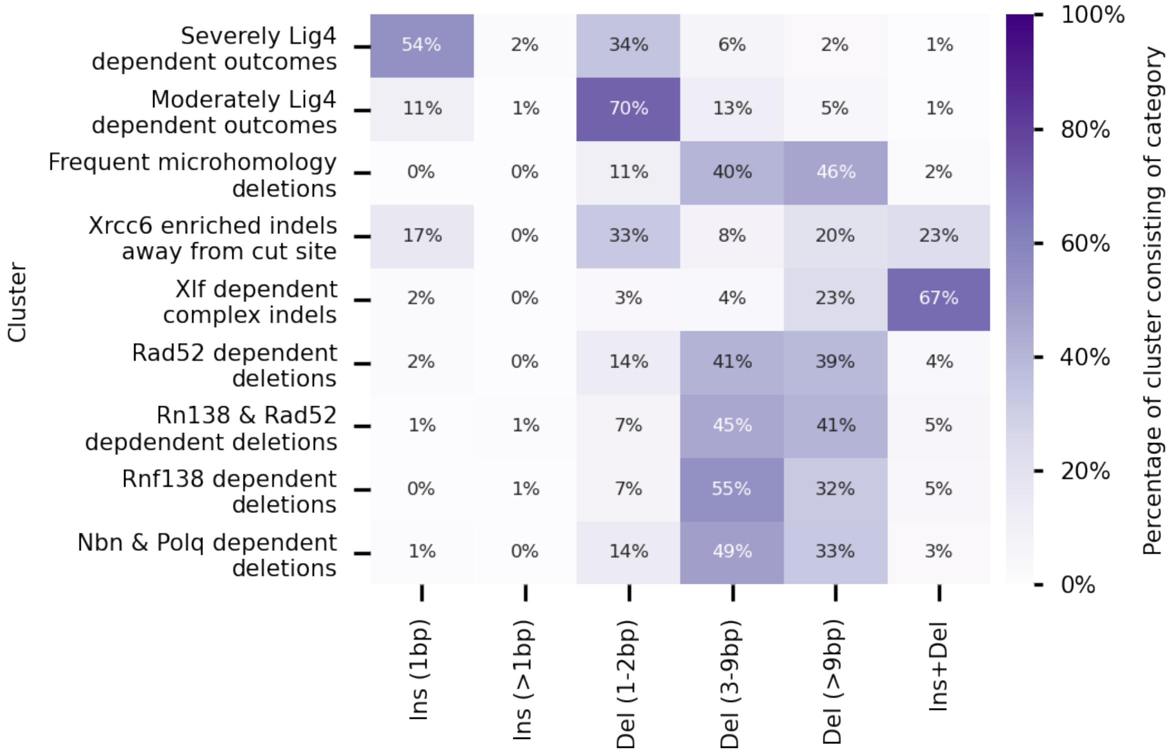

Supplementary Figure 5. UMAP clusters were enriched for particular outcomes categories. Percentage (annotation, colour) of each cluster (y-axis) consisting of each outcome category (x-axis). Each row sums to 100%
